# Particulate Matter Capturing via Naturally Dried ZIF-8/Graphene Aerogels under Harsh Conditions

**DOI:** 10.1016/j.isci.2019.05.024

**Published:** 2019-05-23

**Authors:** Jiajun Mao, Yuxin Tang, Yandong Wang, Jianying Huang, Xiuli Dong, Zhong Chen, Yuekun Lai

**Affiliations:** 1 National Engineering Research Center of Chemical Fertilizer Catalyst (NERC-CFC), College of Chemical Engineering, Fuzhou University, Fuzhou 350116, P. R. China; 2 National Engineering Laboratory for Modern Silk, College of Textile and Clothing Engineering, Soochow University, Suzhou 215123, P. R. China; 3 Institute of Applied Physics and Materials Engineering, University of Macau, Macau, P. R. China; 4 Institute of Functional Nano & Soft Materials (FUNSOM), Jiangsu Key Laboratory for Carbon-Based Functional Materials & Devices, Soochow University, Suzhou 215123, P. R. China; 5 School of Materials Science and Engineering, Nanyang Technological University, 50 Nanyang Avenue, 639798 Singapore, Singapore

**Keywords:** Materials Science, Composite Materials, Nanomaterials, Porous Material

## Abstract

Particulate matter (PM) pollution poses a serious threat to the environment and public health. Capture of PM is best performed at the emission source, such as car exhaust exit points, although it is a challenge for filters to work under harsh conditions of high temperatures and flow rate. Here we designed a thermally stable PM filter by *in situ* anchoring of zeolite imidazole framework-8 (ZIF-8) on a three-dimensional (3D) network of reduced graphene oxide aerogel (rGA) through natural drying. Owing to high specific surface area, well-connected porous network of graphene aerogel, and large number of metal sites from ZIF-8/rGA, the capture efficiencies for PM_2.5_ and PM_10_ are over 99.3% and 99.6%, respectively, at ambient conditions, and the efficiencies remain high in harsh conditions (PM_2.5_ and PM_10_: >98.8% and >99.1%, respectively, at 200°C at a flow velocity of 30 L/min). The filter can be regenerated by a simple washing process.

## Highlights

• ZIF-8/rGA filters are developed by *in situ* anchoring and natural drying strategy
• Filters realize efficient removal efficiency at high temperature and flow velocity
• The robust filters exhibit appealing cycling property for sustainable PM capturing

## Graphical Abstract

**Figure:**
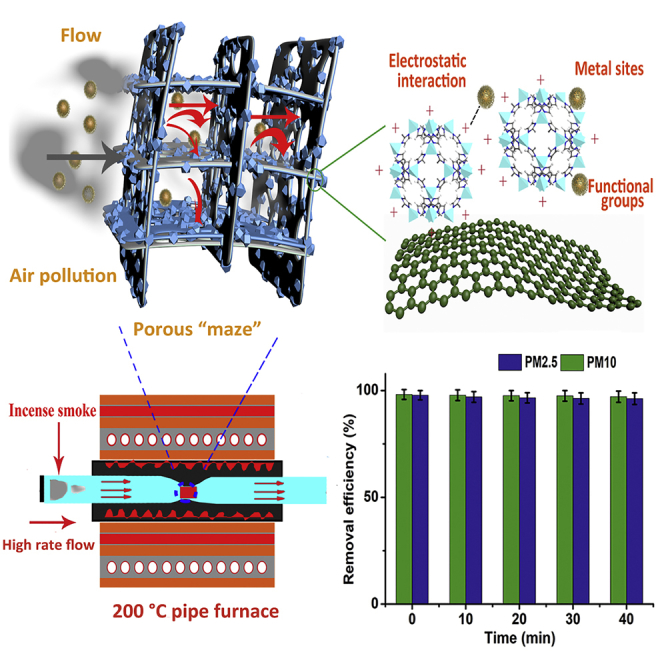

## Introduction

Haze problem, mainly caused by particulate matter (PM)_2.5_ defined by an aerodynamic diameter of 2.5 μm or less, strongly influences human health in our daily life (Gao et al., 2017, Wu et al., 2017). In 2013, 87% of the world population was living in regions that have surpassed the PM_2.5_ limit of 10 μg m^−3^, an air quality limit set by the World Health Organization (He et al., 2015). Especially in countries like China, the average concentrations of PM_2.5_ reached 54.5 μg m^−3^, and 92% of the population has experienced over 120 h of exposure to unhealthy air during a 4-month period (Rohde and Muller, 2015). An analysis of the sources of PM_2.5_ pollution in China indicates that the primary particulate emission comes from traffic, coal or biomass burning, and dust, whereas the secondary origin is mainly from aerosol precursors from SO_x_, NO_x_, and volatile organic compounds (Huang et al., 2014b). PM_2.5_ seriously threatens human health because of its toxic component and the potential menace caused by its small size of penetration of human bronchi and lungs. Based on a report by the Global Burden of Disease, PM_2.5_ was a major predisposing factor for 2.9 million deaths in the 2013 (Ärnlöv, 2015). Therefore highly efficient technology for rapid PM_2.5_ capture, particularly at the source of emission, is urgently needed.

Previous strategies have been explored to tackle PM_2.5_ pollution particles, such as using commercially available air filters (thick fabric, active carbon, etc.), polar polymer nanofiber filters (Khalid et al., 2017, Liu et al., 2015), conductive silver nanowire filters (Jeong et al., 2017), and porous metal-organic frameworks (MOFs) as filters (Chen et al., 2017, Zhang et al., 2016). However, the filtration of conventional air filters is usually performed at a low initial concentration (<1,000 μg cm^−3^) and ambient conditions. For some specific applications, such as the filtration of vehicle exhaust and chimney exhaust, the flow rate and pressure of pollution gas are high under harsh conditions (e.g., high temperature, rapid flow rate, and large humidity). Some particles may not be effectively captured if only these conventional membranes are used. The robust air filters for the realization of both high removal efficiency and low pressure drop for highly concentrated PM under harsh conditions is still a big challenge (Zhao et al., 2017b). Therefore it is desirable to design a 3D porous framework with evenly distributed and well-connected pores for efficient particle capture (Zhang et al., 2018a, Zhang et al., 2018b). An ideal structure should be highly porous to minimize resistance to a high-rate flow gas and possess a large surface area to capture the particles.

Graphene aerogels (GAs), which combine the chemical nature of the material with porous networks, meet the requirements mentioned above. They also exhibit high mass efficiency for separation and adsorption for oils, metal ions, and organic solvents (Bi et al., 2012, Cong et al., 2012). Self-assembly, cross-linking, chemical vapor deposition (CVD), and 3D printing are common methods for the synthesis of GAs (Cao et al., 2011, Wei et al., 2013, Xu et al., 2010, Zhu et al., 2015). Most of these approaches with the exception of CVD involve freeze- or supercritical drying, leading to high cost and low production yield. Compared with these approaches, a natural drying technique is more practical owing to its productivity and good scalability (Li et al., 2016, Xu et al., 2016, Yang et al., 2015). To maximize the removal efficiency using GAs, high-specific-surface-area adsorbents with micro- or nanoscale porosity could be decorated on the GA networks. In this regard, MOFs are ideal candidates as they are ultraporous materials with secondary building units (metal clusters, or known as *metal-containing nodes*) and organic linkers (Furukawa et al., 2013). They have attracted a great deal of interest in energy and environmental fields, such as energy storage, separation, and pollutant control (Sumida et al., 2012, Wang et al., 2014, Zhang et al., 2016).

Here we demonstrate a novel strategy to uniformly decorate MOFs on reduced graphene oxide aerogel (rGA) by the combination of *in situ* crystallization of MOFs and naturally drying the resultant composited hydrogel. The microporous structures of zeolite imidazole framework-8 (ZIF-8) contribute to the high special surface area, whereas the macropores of rGAs provide accessibility to the active surfaces. Free metal sites, functional groups, and electrostatic interaction of ZIF-8/rGAs play the roles of ensuring good filtration efficiency of PM_2.5_. Benefited from these merits, the capture efficiencies for both PM_2.5_ and PM_10_ are over 99.3%, and >99.6%, respectively. The capture efficiencies remain high (>98.6% and 98.9%) after 7-h use. For practical application, we also demonstrated the post-adsorption separation of PM_2.5_ and filter reactivation for reuse, which has been largely neglected in the previous research. This study opens a new avenue for the next-generation filters with 3D advanced networks for fast, efficient, and sustainable treatment of air pollution under harsh working conditions.

## Results and Discussion

### Fabrication of the MOFs/rGA Filter

**Scheme 1:**
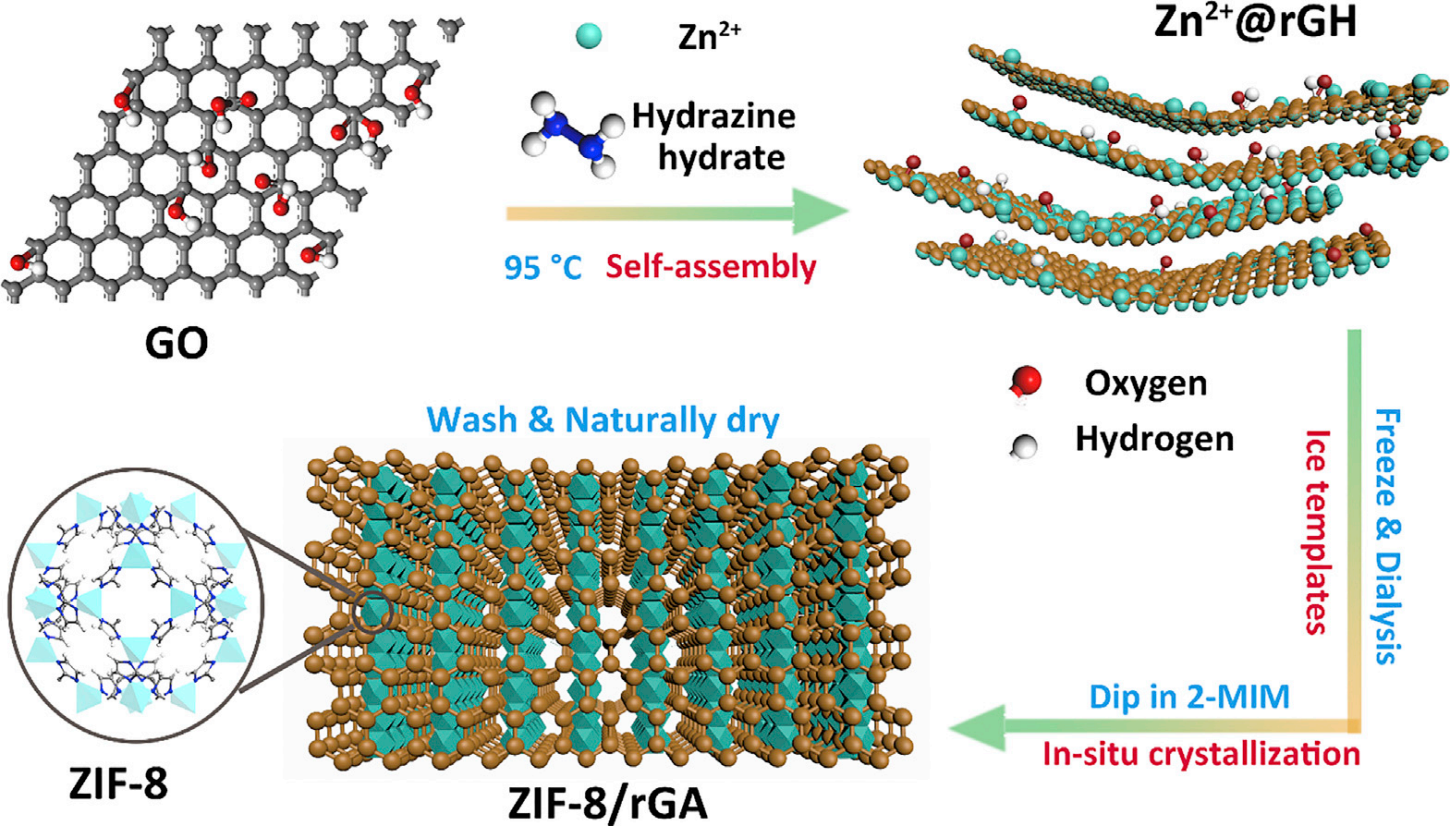
Schematic Illustration of *In Situ* Crystallization of ZIF-8@rGO for the Naturally Dried Aerogel Zn^2+^/reduced graphene oxide hydrogel (rGH) was prepared first by hydrothermal synthesis, followed by the *in situ* crystallization of ZIF-8@rGH by dipping in dimethyl imidazole (2-MIM) and natural drying of the hydrogel after a pre-freeze.

To demonstrate our concept, the filter composited of hybrid ZIF-8/rGA was first synthesized (for detailed information see Transparent Methods). We selected ZIF-8 owing to its low cost, easy synthesis, and well-arranged nano-/microscale surface chemistry (Hu et al., 2016, Huang et al., 2014a). The natural drying method is beneficial for energy and cost considerations. It is also practically important for the scale-up and possible repair after long-term use. The synthesis process of ZIF-8/rGA is illustrated in Scheme 1. Aqueous graphene oxide (GO) was first mixed with zinc nitrate hexahydrate (as the precursors) and hydrazine hydrate (as the chemical reducing agent). Then the suspension was heated at 95°C for 1 h, and it gradually self-assembled into hydrogels due to the abundant oxygen-containing functional groups. The obtained hydrogels were then frozen to prepare the porous frameworks by introducing ice crystals. After being dialyzed with water and methanol in sequence, the excess reagents were removed from the pores. As shown in Figure S1 (Supplemental Information), various shapes of hydrogels could be synthetized when containers of different shapes were utilized. After the hydrogel formation, the sample was dipped in methanol solution of 2-methylimidazole during which ZIF-8 was formed *in situ*. After washing and drying in air, ZIF-8/rGAs were obtained with a density of ca. 10–15 mg cm^−3^. The aerogel prepared through the ambient drying technique showed no obvious volume shrinkage and is able to maintain structural integrity (Figure S2A). This is because the ice template forms a robust network that possesses a strong resistance to the capillary force during the evaporation of solvent (Xu et al., 2016). Meanwhile, the low freezing temperature (−80°C) with rapid cooling rate has resulted in a honeycomb-like porous structure with nanopores (Deville et al., 2006, Pawelec et al., 2014). When different amount of graphene was added, the density of the aerogel varied accordingly (Figure S2B).

**Figure 1:**
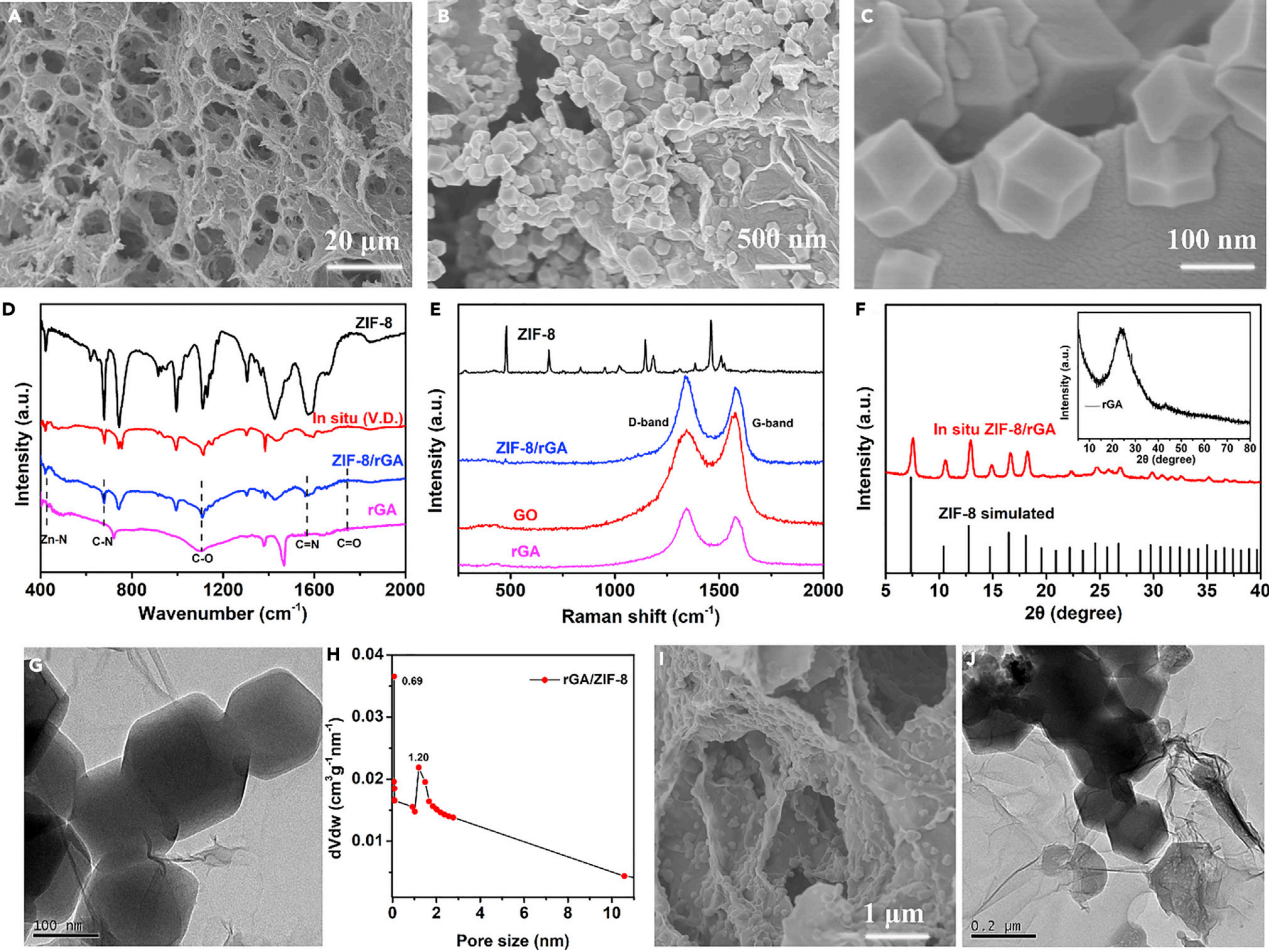
Characterization of As-Prepared ZIF-8/rGA (A–J) (A–C) SEM images of ZIF-8/rGA obtained at different magnifications. (D and E) (D) FTIR and (E) Raman spectra of rGA, ZIF-8, ZIF-8/rGA by blending, and *in situ* ZIF-8/rGA; the D and G bands are characteristic of rGO. (F) XRD patterns of the rGA and *in situ* ZIF-8/rGA. (G) TEM image of *in situ* ZIF-8/rGA. (H) Barrett, Joyner and Halenda (BJH) pore size distributions of *in situ* ZIF-8/rGA. (I) SEM and (J) TEM images of the cross section of *in situ* ZIF-8/rGA.

As demonstrated by scanning electron microscopy (SEM) and transmission electron microscopy (TEM), the typical as-synthesized ZIF-8/rGAs showed 3D interconnected porous networks with continuous macropores (Figure 1A). ZIF-8 particles were homogeneously decorated on the surface of rGAs with size ranging from 80 to 200 nm (Figures 1B and 1C). It is worth noting that ZIF-8 particles were uniformly encapsulated within networks, as evidenced by the cross-sectional SEM and TEM images (Figures 1I and 1J). The open and well-connected porous structure enables maximum surface interaction with the PM particles and thus enhances the adsorption efficiency. Caution should be exercised on the amount of Zn precursor added during preparation: too small amount leads to uneven distribution of MOFs, whereas too large amount causes excessive MOF particles (Figures S3A and S3B).

Peaks from Zn-O, C-N, and C=N bonds were observed in Fourier transform infrared (FTIR) spectra and gave evidence of successful *in situ* formation of the ZIF-8 (Figure 1D). In the Raman spectra (Figure 1E), the D- and G-bands of GO and ZIF-8/rGA appeared after the self-assembly processes, and the increase of D/G intensity ratio indicates the reduction of GO (Stankovich et al., 2007). As shown in Figures 1F and 1G, the X-ray diffraction (XRD) and the TEM studies further confirmed the *in situ* decoration of ZIF-8 particles in the composite (Park et al., 2006). However, there was no apparent diffraction peak around 25° for graphene, indicating that the efficient loading of ZIF-8 on the surface has suppressed the stacking of rGA sheets. Besides, N_2_ adsorption-desorption isotherms show various cumulative volumes of pores (Figure 1H), and N_2_ adsorption-desorption analysis shows that the prominent pore size in the ZIF-8/rGA was from 0.7 to 2.7 nm.

**Figure 2:**
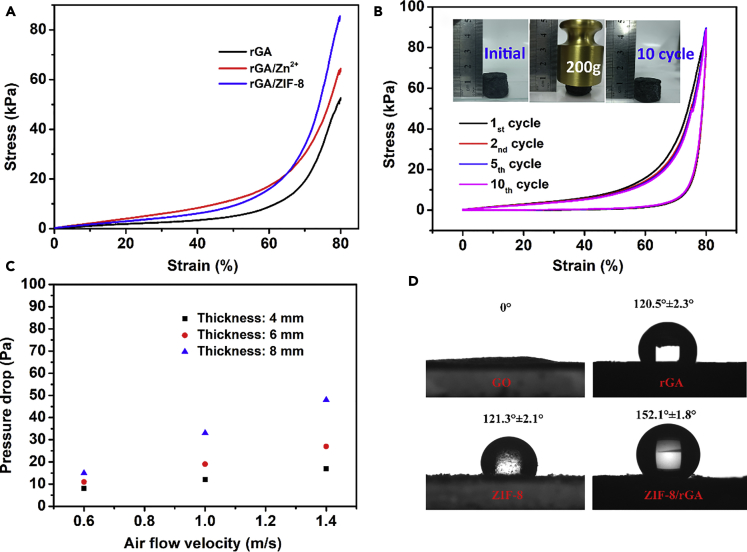
Performance of As-Prepared ZIF-8/rGA (A and B) (A) Stress-strain curves of rGA, Zn^2+^/rGA, and rGA/ZIF-8, (B) Cyclic compressive stress-strain curves of ZIF-8/rGA; inset shows ZIF-8/rGA before and after supporting 200 g weight. (C) Pressure drops of the ZIF-8/rGA aerogel filter with different thicknesses at different air flow velocities. (D) Water contact angle measurements of pristine GO, rGA, ZIF-8, and ZIF-8/rGA samples.

In addition, the prepared samples displayed high thermal stability and mechanical strength. The thermal stability of ZIF-8/rGA was investigated by thermogravimetric analysis (TGA) measurements (Figure S4). The aerogel was thermally stable for up to 200°C, and this could be in favor of well-retained PM removal efficiency at high working temperatures. The stress-strain curves of GA, Zn^2+^/rGA, and ZIF-8/rGA show that ZIF-8/rGA has better mechanical performance (Figure 2A). Zn ions in the MOF could serve as the cross-linkers for the promotion of ZIF-8/rGA skeleton stiffness. Furthermore, naturally dried ZIF-8/rGA possesses superelasticity and good fatigue resistance (Figure 2B). This is a result of smaller pore sizes and noticeable squeezing effect of ice-crystal among two orthogonal directions, emerging from the much thinner ice crystals and rapid crystal growth rate at low temperature (Gao et al., 2016, Yang et al., 2015). However, the aerogel is relatively easy to be damaged from the perpendicular directions, where the lamellar is not preferentially aligned in a single direction. As shown in Figure 2C, the pressure drop of the as-prepared aerogel filter is as low as 22 Pa (filter thickness = 4 mm) at the air flow velocity of 1.4 m/s.

### Performance of Uniformly Decorated ZIF-8/rGA Filter

**Figure 3:**
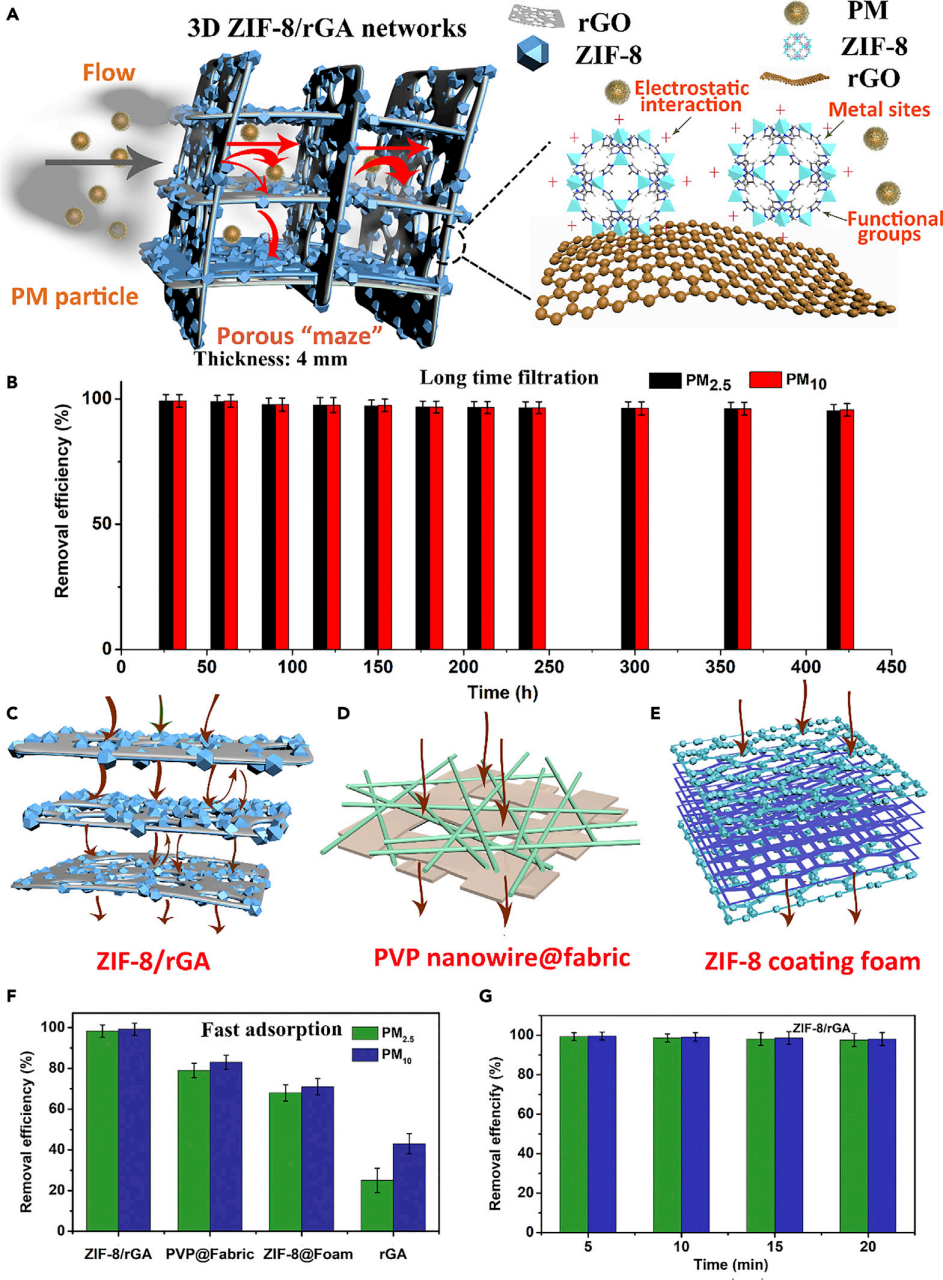
Comparison of PM Removal Properties of Diverse Samples (A and B) (A) Proposed capture mechanism of 3D networks of ZIF-8/rGA and (B) long-time PM removal efficiency measurement (error bars represent the standard deviation among three parallel tests). Schematic illustration of fast microparticle collection process when PM passes through various filters with functional co-continuity networks. (C–E) (C) ZIF-8/rGA (4 mm), (D) 2D polymer nanofiber filter (0.5 mm), and (E) ZIF-8 coating foam (4 mm, most of the ZIF-8 particles decorate on the outermost surface of the foam). (F and G) (F) Fast adsorption performance of diverse filters and (G) stability performance of ZIF-8/rGA during fast adsorption.

Primary and secondary particulate emissions from car and stack exhaust occupy a large portion of PM_2.5_; suppressing the particle pollution from the source is a significant way to control air pollution (Han et al., 2015). PM removal efficiency was investigated with a home-built device (Figure S5) under ambient condition. The long-term performance is vital for the practical application of ZIF-8/rGA in real environment. Here incense burning was employed to simulate the exhaust emission. The burning incense generated particles with a broad size distribution ranged from 11.1 to 1,083.3 nm, consisting of carbonyls, polycyclic aromatic hydrocarbon, azoarenes, and metallic compounds (Glytsos et al., 2010, Lui et al., 2016). Figure 3A illustrated that ZIF-8/rGA serves as an open-wall honeycomb-like filter for PM capturing. When the exhaust gas entered the open pores, most of PM particles would be trapped by the 3D networks of ZIF-8/rGA, which possess tortuous and discontinuous macrochannels. For the ZIF-8, the PM particle cannot enter inside the pores of ZIF-8 because the size of the PM particles is larger than the pore size. The fine nanoparticles can be captured on the surface of ZIF-8 (Figure S6) owing to the effects of electrostatic interactions, surface functional groups, and numerous metal sites (Chen et al., 2017, Zhang et al., 2016).

**Figure 4:**
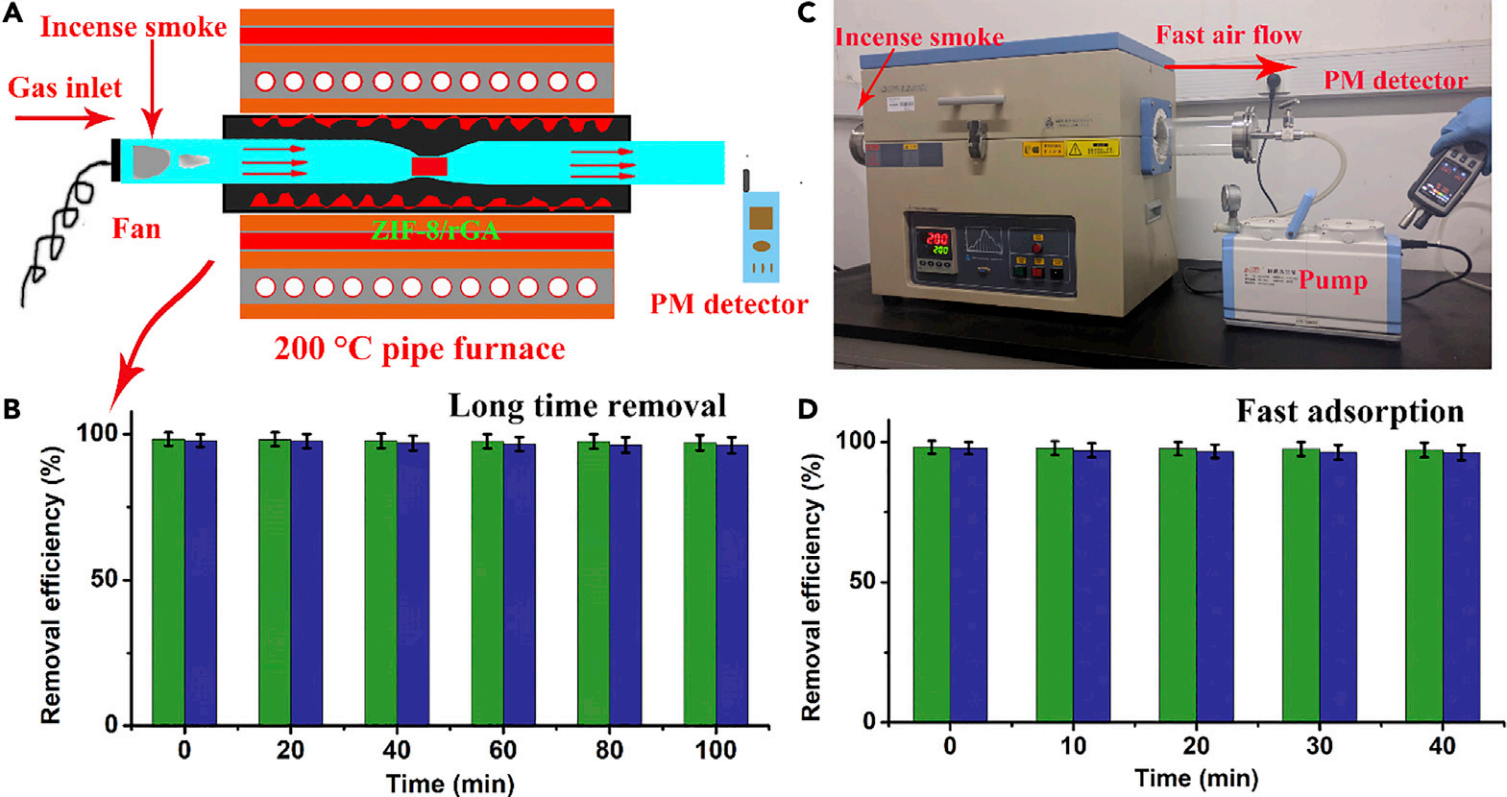
PM Capturing at High Temperature (A–D) (A) Schematic illustration of modified pipe furnace for high-temperature PM removal test, (B) long-time PM removal efficiency, (C) photograph of the filter place in pipe, and (D) fast PM removal test under temperature of 200°C.

PM removal efficiency was investigated in a home-built device (Figure S5) under ambient condition. All the ZIF-8/rGA filters are aggregated to uniform size with 1 cm thickness and 1 cm radius. Removal of both PM_2.5_ and PM_10_ was maintained at a high level (>98.6%) after a 7-h test while a great deal of particle pollution (PM_2.5_ > 1,500 μg m^−3^ and PM_10_ > 2,000 μg m^−3^) was generated from incense smoke (Figure 3B). The weight of aerogel increased by 3.8%. SEM image (Figure S6) shows that the pollution particles were uniformly covering the MOF surface, suggesting a superior capture efficiency of ZIF-8/rGA. Functional groups, open metal sites, and electrostatic interaction make MOFs promising candidates for pollutant removal. In addition, rGA not only severs as a skeleton but also provides a large amount of porous contact area to interact with PM particles. While air pollutant particles pass through this layer-by-layer porous framework, the continuous lamellar structure ensures sufficient opportunities for the particles to interact with the adsorbent. On the other hand, pure rGA displayed poor efficiency of PM removal (PM_2.5_: 40.2% ± 3.4%, PM_10_: 58.4% ± 2.8%, Figure S7). The grafting of ZIF-8 on graphene matrix has clearly played an important role in the improved removal efficiency (Jung et al., 2018). With increasing ZIF-8 loading, removal efficiency has increased from 76.1% to 99.3%. However, there is a saturation point, and excessive ZIF-8 is not helpful (Figure S3C). Moreover, compared with the composite aerogel filter constructed by directly blending of ZIF-8 and GO in water solution, the *in situ*-grown ZIF-8/rGA aerogel filter demonstrated a higher efficiency. This could be ascribed to the uniform decoration of MOFs with numerous functional sites on graphene layers and its higher specific surface area (the mixed ZIF-8-rGA possesses a lower Brunauer–Emmett–Teller (BET) of about 165 m^2^ g^−1^, Figure S8). A modified tube furnace that provides gas flowing at high temperature was used to simulate PM removal under harsh conditions (Figure 4A). ZIF-8/rGA filter shows superior PM removal efficiency at 200°C (PM_2.5_: 98.9% ± 1.6%, PM_10_: 98.6% ± 1.8%) and well-retained removal efficiency after 100 min (PM_2.5_: 98.3% ± 1.6%, PM_10_: 97.8% ± 1.8%) in Figure 4B. Two-dimensional filters could effectively remove PM pollution from the sources (vehicle and stack) and prevent outdoor pollution invading indoor system or human body; however, they have a poor performance in the fast adsorption of PM pollution (Zhang et al., 2017). Here, a diaphragm pump was designed to evaluate the ability of the ZIF-8/rGA aerogel filter to continuously collect PM particles. The pump was connected with smoke source, and the entire collecting process would not stop until all the PM was cleaned away.

### Efficient Removal of PM under a Large Velocity of Air Flow

Maintaining high-efficiency PM filter under rapid air flow removal is significant (He et al., 2016). A highly sensitive particle detector was used to test if PM pollution “evades” through the filters during the rapid removal process. Figures 3C–3F displayed the schematic illustration and investigated the efficiency of four types of filters (*in situ* ZIF-8/rGA, thickness: 4 mm, polyvinylpyrrolidone [PVP] nanowire@fabric, thickness: 0.5 mm, ZIF-8 coating melamine foam, thickness: 4 mm, and pristine rGA, thickness: 4 mm) under the assistance of air pump with a flow rate of approximately 30 L min^−1^. All the filters are chosen with a close pressure drop; the aerogel with the through-pores exhibits less pressure drop even with larger thickness. Detailed preparation methods are described in Transparent Methods. The PM removal efficiencies of these filters were 99.1% (PM_2.5_) and 99.3% (PM_10_) for ZIF-8/rGA (with Zn^2+^:GO of 3:1), 81.9% (PM_2.5_) and 82.3% (PM_10_) for PVP@fabric (Figure S9), 75.5% (PM_2.5_) and 77.3% (PM_10_) for melamine foam with ZIF-8, and 38.5% (PM_2.5_) and 63.2% (PM_10_) for rGA. As air pollution passes through the ZIF-8/rGA filters, the well-connected microporous structure ensures high air flow and enhances the particle penetration into the filter (Figure S10). Microporous ZIF-8 uniformly decorated on all stacked graphene layers is responsible for the high-efficiency binding for both PM_2.5_ and PM_10_ by electrostatic interactions and through the numerous active functional sites. Mass PM removal efficiency ratio of ZIF-8/rGA was superior to that of filters with low effective mass loading (PVP nanowire@fabric and ZIF-8 coating foam). Moreover, as shown in Figure 3G the ZIF-8/rGA filters maintained a high PM_2.5_ and PM_10_ removal efficiency during the entire process of PM particle collection. This experiment verified that the composite ZIF-8/rGA could effectively resolve the PM leakage problem in the conventional filters, which makes it a significant potential candidate for practical application. Here we compared the pressure drop and removal efficiency of diverse materials in the large air flow condition (Figure S11). The PVP filters with larger thickness are obtained by different numbers of stacked PVP@fabric. Even the PVP nanowire film could achieve higher removal efficiency with larger thickness; its pressure drop sharply increases to a high value (∼2,256 Pa of 4 mm). ZIF-8@foam filters express low pressure (∼164 Pa of 4 mm), but their removal efficiency also maintains relatively low levels. Owing to the synthetic effect of continuous networks of rGA and numerous sites of MOFs, rGA/ZIF-8 obtains ∼99% removal efficiency at low pressure drop (∼158 Pa of 4 mm).

**Figure 5:**
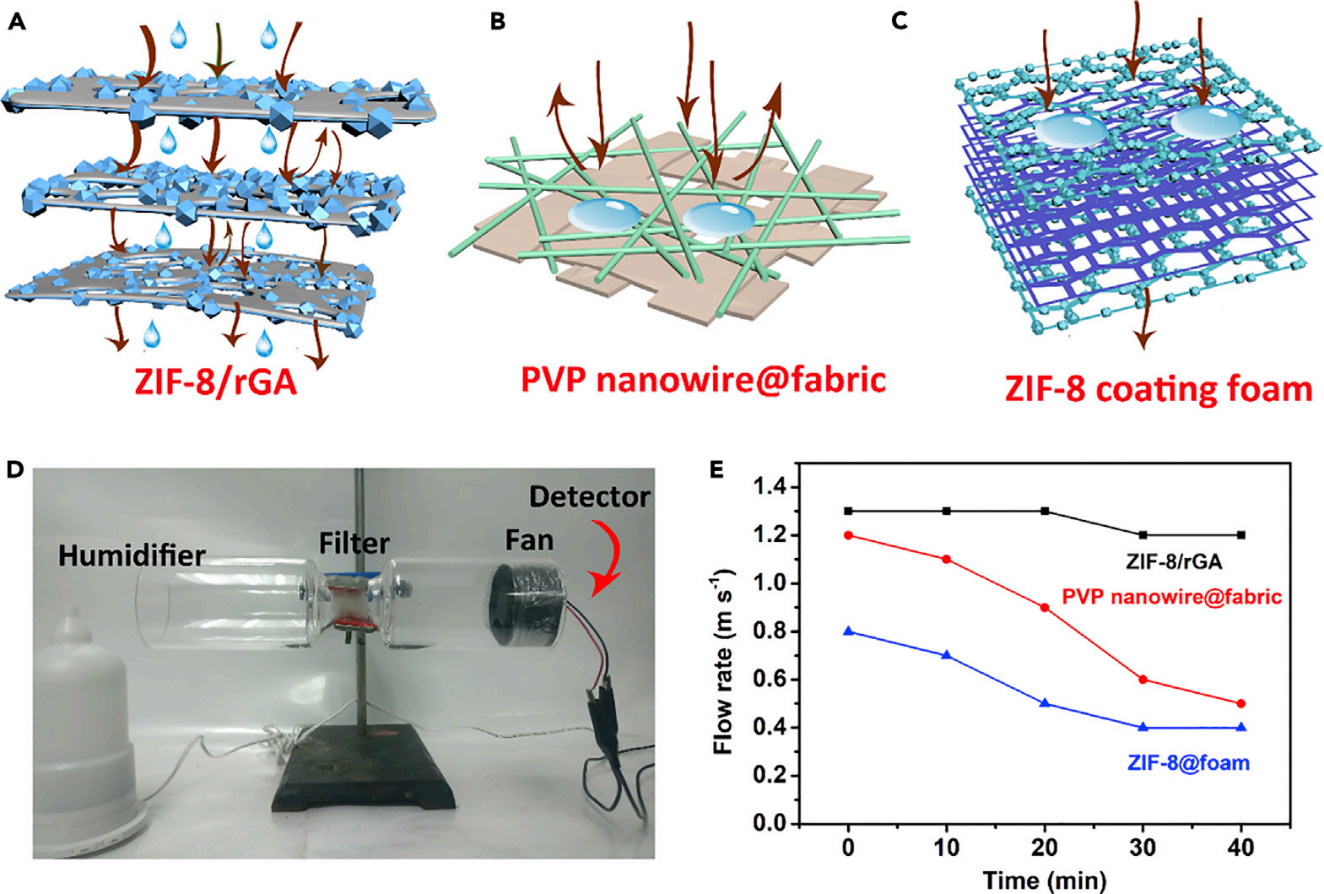
Air Permeability of Diverse Samples under Humidity Schematic illustration of air flow pass through various filters under humidity conditions. (A–C) (A) ZIF-8/rGA (4 mm), (B) 2D polymer nanofiber filter (0.5 mm), and (C) ZIF-8 coating foam (4 mm). (D) Photograph of wetted air flow pass through various filters. (E) Air permeability test of filters at different times under humidity conditions.

Diaphragm pump has also been applied at high temperature for fast PM removal (Figure 4C). The PM concentrations before and after filtration were calculated in Figure 4D; the ZIF-8/rGA filter can effectively remove PM particles (PM_2.5_: >99.1% and PM_10_: >98.8% after 40 min) even under a high temperature of 200°C and high pressure. The rapid and high-efficiency PM removal under high-temperature environment is significant for managing industrial and vehicle exhaust gas. In addition, vehicle exhaust gas produces water vapor that may accumulate and block the pores of filters (Zhao et al., 2017a). ZIF-8/rGA exhibited superhydrophobicity with a contact angle of 152° due to the low surface energy rGO and micro-/nanoscale hierarchical rough structure (Figure 2D). The water repellent property of ZIF-8/rGA facilitates fast mobilization of water and ensures a high air flow rate (Figures 5A–5C). An anemograph (EDKORS, FS-801) was applied to detect gas flowing at a constant wind velocity under certain humidity condition (Figure 5D); ZIF-8/rGA (4 mm) maintained high air permeability during long-time removal, but PVP nanowire@fabric and ZIF-8@foam (4 mm) display an obvious air permeability decline after 30 min as a result of blocking effect of water vapor on hydrophilic filters. In Figure 5E, the air flow rate was measured to be 1.2 m s^−1^ and 1.4 m s^−1^ with and without ZIF-8/rGA, respectively. This pressure drop was small, indicating an excellent air flow penetration for high-efficiency PM removal. Moreover, the loading and distribution of MOF particles in the foam scarcely affects the air permeability.

### The Characterization of PM Adsorbed on the Filter

**Figure 6:**
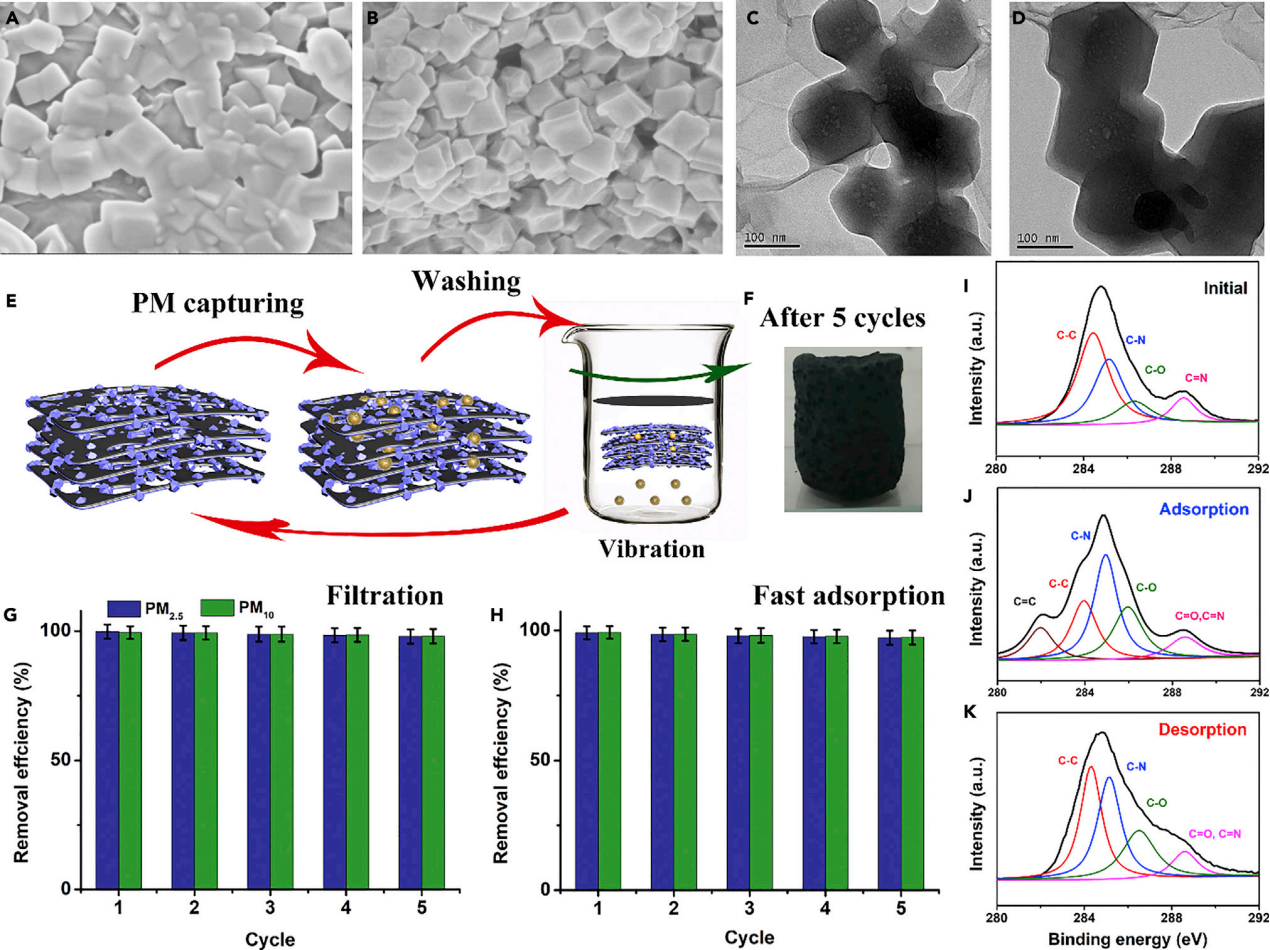
Characterization of PM@ZIF-8/rGA and after Washing (A–D) (A and B) SEM images of PM attached on ZIF-8/rGA and washed away from the aerogel. (C and D) TEM images of PM pollution absorbed on the surface of the ZIF-8/rGA and washed away. (E–H) (E) Schematic diagram of recycling process, (F) photograph of ZIF-8/rGA after five cycles of PM removal test, and (G and H) cycle performance of ZIF-8/rGA in (G) filtration and (H) fast adsorption. (I–K) High-resolution C 1s peak analysis of the composition of 3D networks (I) before removing PM pollution, (J) after PM adsorption, and (K) after PM desorption by washing successively in ethanol and water solution.

**Figure 7:**
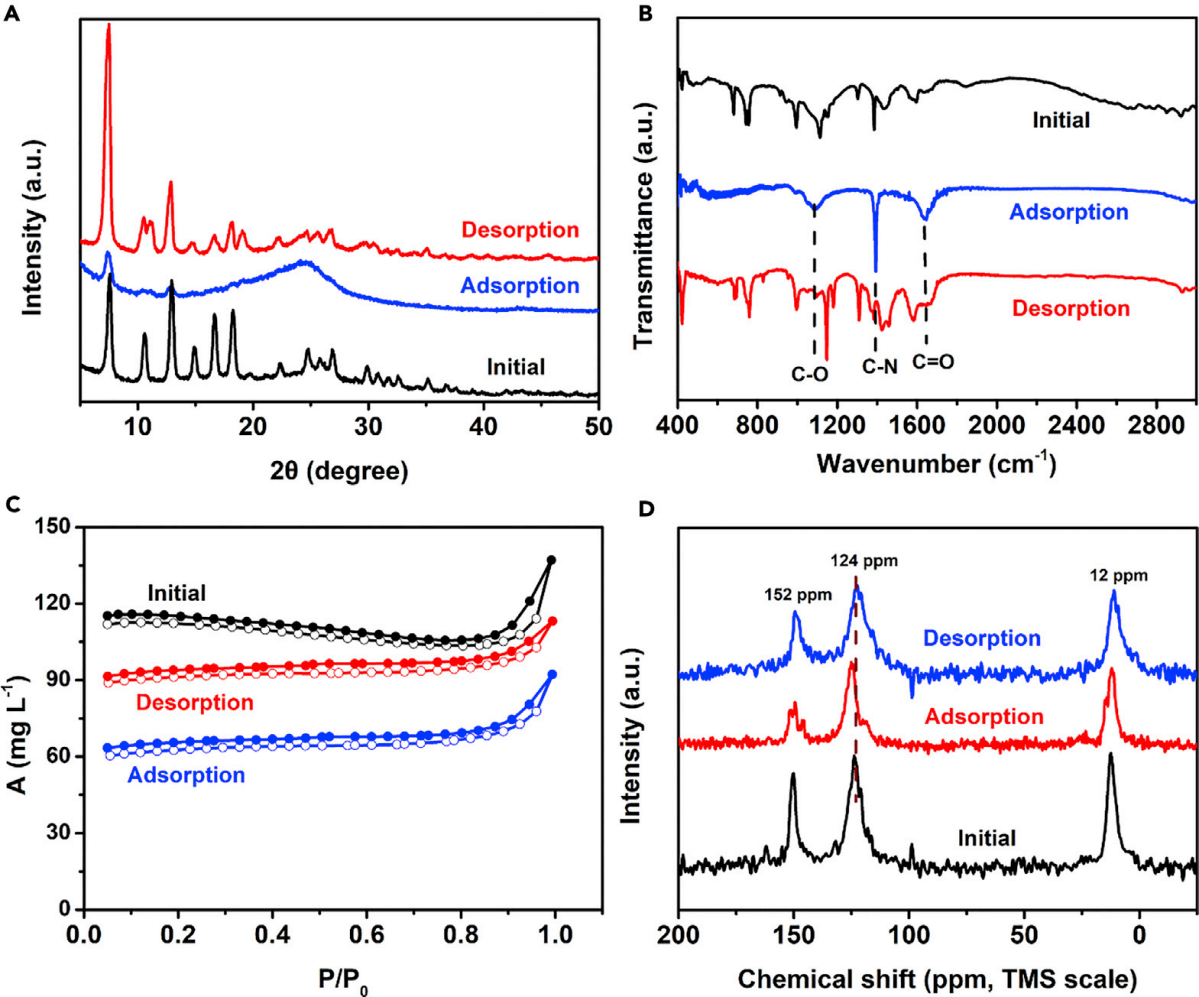
Composition Analysis of Aerogel during PM Capturing (A and B) The composition analysis of 3D networks before removing PM pollution, during adsorption of PM, and during washing in ethanol with water. (A) XRD spectra and (B) FTIR spectra. (C and D) (C) N_2_ isotherms at 77 K and (D) solid-state ^13^C nuclear magnetic resonance spectra of initial aerogel, PM adsorbed on aerogel, aerogel recycled by washing.

To further explore the performance of ZIF-8/rGA in capturing PM pollution, the surface chemistry and chemical composition of PM adsorbed on the aerogel was investigated. Figures 6A–6D show the SEM and TEM images of PM attached to the ZIF-8/rGA; the particles have a sticky amorphous carbon-like morphology. Some nanosized PM particles uniformly bind to the ZIF-8 surfaces, whereas PM with the microsized PM particles randomly distribute on the lamellar surface. The XRD pattern of the PM particles shows a broad peak around 25°, suggesting that the PM particles are amorphous (Figure 7A). The fact that the characteristic peaks of ZIF-8 were suppressed indicates that the PM particles have fully covered the ZIF-8 surface. The surface chemistry was investigated by FTIR (Figure 7B). The spectra only showed functional groups C-O, C-N, and C-C with peaks at ∼1,075, 1,120, and 1,645 cm^−1^ after PM adsorption. These expressed groups are consistent with PM particles adsorbed on the aerogel. XPS images shown in Figures 6I–6K only examine the surface element of the aerogel. The C 1s peaks are composed of five signals corresponding to C=C, C-C, C-O, C-N, and C=O bonds. The ratios of functional groups C=C, C-C, C-O, and C-N on the as-prepared aerogel, PM absorbed aerogel, and PM-desorbed aerogel are about 0:4.3:3.6:1, 0.7:1:1.9:1, and 0:3.5:3.2:1, respectively. The results indicate an increase of the C-N, C=C, and C-O after PM removal. The three elements present are consistent with measurements by energy-dispersive spectrometry (Table S1). Textural parameters such as surface area initially, before, and after PM removal was obtained by N_2_ sorption isotherms at 77 K (Figure 7C). The BET surface areas of the hybrid aerogel initially, before, and after PM removal were 330.79, 195.06, and 282.50 m^2^ g^−1^, respectively. After adsorbing PM particles, the special surface area has a sharp drop due to particle pollution partially blocking the micropores of ZIF-8. The typical surface morphology of the aerogel is recoverable through water and ethanol vibration washing. TGA result (Figure S4) revealed obvious differences between pure ZIF-8/rGA and PM attached on ZIF-8/rGA from 140°C to 270°C as a result of absorbed unreacted molecules. Figure 7D displays the ^13^C nuclear magnetic resonance spectra of pure ZIF-8/rGA, PM attached on the ZIF-8/rGA, and recycled ZIF-8/rGA. Although the signal of methyl groups from ZIF-8 does not shift, unoxidized double carbon bond (124 ppm) undergoes a noticeable change due to hydrogen bond from hybrid aerogel interaction with oxygen-containing groups in particle pollution. Furthermore, carbon atom between the two nitrogen atoms of the imidazolate rings (152 ppm) presents three individual resonances while PM is attached on it and indicates a lower symmetry than pure ZIF-8/rGA in the orthorhombic space group.

The polarity of the hybrid aerogel was detected by zeta potential, and the as-synthesized networks displayed large cavities with narrow windows and a high zeta potential. Besides, PM pollutant particles express highly polarity due to the existence of moisture and polar functional groups. The unbalanced metal ions on the surface of MOFs offer the positive charge and thus can polarize the surface of PM improving the electrostatic interactions. After a 7 h PM removal, we examine the zeta potential of PM@ZIF-8/rGA which still maintain a high zeta potential (>100 mV), and this indicates electrostatic interactions should play a vital role in long time PM removal. According to our measurement, we proposed that the unbalanced metal ions on the surface of MOFs offer positive charge and thus can polarize the surface of PM improving the electrostatic interactions. Meanwhile, the ability to maintain high air flow is also a significant parameter for the removal performance of a filter (Huang et al., 2019). We evaluate air permeability by measuring the permeation of ammonia sealed by the hybrid aerogel (Figure S10C). The color of the pH indictor turned purple as soon as the indictor was placed near the aerogel.

### Cycle Performance of ZIF-8/rGA Filters

Rational air pollution control strategy requires not only the efficient removal of pollutants and the effective prevention of secondary environment pollution but also easy recycling and reuse of the filter (Ding et al., 2016). The recycle performance and the regeneration of ZIF-8/rGA are illustrated in Figures 6E–6H. The recycling process could be achieved by washing by ethanol and water with mild agitation, followed by natural drying. The majority of PM absorbed onto the ZIF-8/rGA filter was able to be washed away as displayed in Figure 6E; meanwhile ZIF-8 resumed dodecahedron structure as shown in Figure 6F. SEM and TEM images obviously displayed the particles have been washed away from the hybrid aerogels. This hybrid filter showed negligible structure and mass change (<0.3 wt %) after 5 cycles. In addition, both XRD and FTIR of ZIF-8/rGA after washing indicated that the skeleton maintains its chemical constituents, functional groups, and crystal form. On the other hand, polarity and specific surface area of aerogel are significant parameters for removal efficiency. The washed 3D network retains a high level of cavities and high PM removal efficiency for five cycles in both simulated haze environment (PM_2.5_: 98.2% and PM_10_: 98.4%, Figure 6G) and the fast flow test (PM_2.5_: 97.9% and PM_10_: 98.1%, Figure 6H).

### Conclusions

Versatile and scalable ZIF-8/rGA filters were successfully constructed via an *in situ* growth process through natural drying. Low freezing temperature induces numerous small pores and robust networks and minimizes the volume shrinkage and structure cracking during ambient drying process. ZIF-8/rGA exhibited a high special surface area, 3D connected structure, and numerous functional groups, making it an ideal candidate for cost-effective PM filter. The optimal 3D ZIF-8/rGA filters exhibited superior capture efficiency (PM_2.5_: >99.3%, and PM_10_ > 99.6%) and good cycle performance (>98.9% after 5 cycles). In addition, they displayed high PM capturing efficiency (PM_2.5_: >98.9% and PM_10_: >98.8%, after 40 min) under harsh working conditions (high flow rate of 30 L min^−1^ and a temperature of 200°C). Systematic investigation confirms the key role of the specific 3D porous maze structure and uniform ZIF-8 particles in the process of PM particle capturing. This study opens a new avenue for the next-generation filters for fast, efficient, and sustainable treatment of air pollution under harsh working conditions.

### Limitations of Study

In this work, we designed ZIF-8/rGA filters for rapid and efficient PM removing under harsh conditions. However, the high temperature, rapid air flow, and large humidity conditions are simulated at the laboratory. It would be more interesting if the aerogel is enlarged and measured *in situ* in the real car exhaust.

## Methods

All methods can be found in the accompanying Transparent Methods supplemental file.

## Supplemental Information

Document S1. Transparent Methods, Figures S1–S11, and Table S1
