# Supplementary material for: Particulate Matter Capturing via Naturally Dried ZIF-8/Graphene Aerogels under Harsh Conditions
Source: iScience. 2019 May 23;16:133–44. doi: 10.1016/j.isci.2019.05.024 (PMC6551531; doi:10.1016/j.isci.2019.05.024)
Supplement: Document S1. Transparent Methods, Figures S1–S11, and Table S1 [file mmc1.pdf]

ISCI, Volume *16*

Supplemental Information

Particulate Matter Capturing via Naturally Dried ZIF-8/Graphene Aerogels under Harsh Conditions

Jiajun Mao, Yuxin Tang, Yandong Wang, Jianying Huang, Xiuli Dong, Zhong Chen, and Yuekun Lai

Transparent Methods

Synthesis of GO, rGA, ZIF-8, ZIF-8/rGA: GO was prepared by modified Hummers' method. ZIF-8: 0.256 g $Zn(NO_3)_2$ $6H_2O$ was dissolved in 25 mL methanol (denoted as solution A) and 0.56 g 2-methylimidazole was dissolved in another 25 mL methanol (denoted as solution B), then solution A was slowly poured into solution B under stirring, after 24 h at room temperature, the solution was washed with methanol three times for products. ZIF-8/rGA: 12 mL of 1 mg mL^{-1} GO aqueous solution and 12 μL hydrazine hydrate were mixed in a cylindrical sample vial, and the solution of 0.8 mL MOFs (mass ratio 1 : 2) earlier synthesized was poured into the mixture. The vessel was then placed into an electrical oven at 95 ℃ for 1 h. Ultimately, the 3D hydrogel was taken out and washed with deionized water and alcohol, then freeze-dried into an aerogel. For comparison, rGA was fabricated by adding reductant only.

In-situ ZIF-8/rGA by natural drying: In a typical synthesis process, 10 mL of 3.0 mg mL^{-1} GO aqueous solution, 40 μL sodium dodecyl sulphate solution (5 wt%), and 30 μL hydrazine hydrate mixed in cylindrical sample vial, and 80 mg $Zn(NO_3)_2{\bullet}6H_2O$ was instilled in the mixture. The vessel was placed in an electrical oven at 95 ℃ for 1 h and the 3D hydrogel was taken out to be freezed in -80 ℃. Then hydrogel was deionized in water, alcohol, and dipped in 10 mL dimethyl imidazole solution (containing 300 mg dimethyl imidazole). Ultimately, the hydrogel was washed by methanol and water and naturally dried in ambient condition.

Synthesis of PVP nanowire@fabric: 7 wt% polyvinylpyrrolidone in ethanol was loaded in a 1 ml syringe with a 22-gauge needle tip, which is connected to a voltage supply. The pure fabric was used to collect the electrospun nanofibres.

Synthesis of ZIF-8 coating melamine foam: The synthetized ZIF-8 was roll-to-roll hot-pressed on melamine foam.

Characterizations: The morphology of aerogel surface was observed by field emission scanning electron microscope (Hitachi S-4800) at 3.0 kV. An energy dispersive X-ray spectrometer fitted to the SEM was applied for elemental analysis. A Kratos Axis-Ultra HSA X-ray photoelectron spectrometer was used to examine the chemical composition with a 100 W $Al_{K\alpha}$ X-ray source and a base pressure of about 4.0×10^{-9} mbar. The volumes of droplets used for the static contact angle measurement were 6.0 μL. The crystal phases of the samples were characterized by an X-ray diffractometer with $Cu_{K\alpha}$ radiation (Philips, X'pert-Pro MRD). The Raman spectra were analyzed by A Raman spectrometer (HORIBA JOBIN YVON, HR800). Fourier transform infrared spectra were obtained on a Varian Scimitar 1000 spectrophotometer with a scan range of 4000-400 cm^{-1}. Transmission electron microscopy was detected on a Transmission electron microscope (FEI Tecnai G-20). Zeta potentials were measured on a Malvern Zetasizer Nano ZS90, the aerogel were dispersed in ethanol and sonicated for several minutes before measurement. N_2 sorption isotherms were measured at a Surface area and porosity analyser (ASAP-2020).

Filtration of PM pollution by ZIF-8/rGA: The filtration of PM pollution of the aerogel was measured in the home-built device shown in Figure S5b, and the device was put in

a smoke environment among the measurement. A ZIF-8/rGA filter was set at middle the case, and an electric fan is put on the terminal to help the air pass through the filter. The filtered air was collected in a plastic bag behind fan, and a highly sensitive particle counter (CEM DT-9880M) was applied to test the PM mass concentrations with and without the filter. The detection was finished before the plastic bag reached its maximum volume.

Fast adsorption of PM pollution by ZIF-8/rGA: The fast adsorption of PM pollution of the aerogel was measured in the home-built device shown in Figure S5c. One side of a peristaltic pump was connected with a container filled with PM pollution by incense smoke, the other side was connected with another container to collect he filtered air. A particle counter (CEM DT-9880M) was applied to test the PM mass concentrations after the adsorption.

Filtration of PM pollution under high temperatures: The filtration of PM pollution of the aerogel was tested in a modified tube furnace shown in Figure 4 at 200 ℃. A ZIF-8/rGA filter was set at middle in the case, and an electric fan or pump is put on the terminal to help the air pass through the filter. The filtered air was collected in a plastic bag behind fan, and a highly sensitive particle counter (CEM DT-9880M) was applied to test the PM mass concentrations with and without the filter. The detection was finished before the plastic bag reached its maximum volume.

Table S1. Data are represented as EDS analysis, related to Figure 5. EDS spectrum of (a) initial ZIF-8/rGA, (b) PM attached on ZIF-8/rGA, (c) PM@ZIF-8/rGA after washing.

a

Element	Weight %	Weight % σ	Atomic %
Carbon	59.862	1.967	73.208
Nitrogen	17.247	2.548	18.087
Oxygen	5.136	0.679	4.716
Zinc	17.755	0.726	3.990

b

Element	Weight %	Weight % σ	Atomic %
Carbon	55.957	2.599	66.568
Nitrogen	22.216	3.364	22.663
Oxygen	8.894	1.130	7.943
Zinc	12.932	0.807	2.827

c

Element	Weight %	Weight % σ	Atomic %
Carbon	61.032	1.986	73.537
Nitrogen	17.000	2.534	17.564
Oxygen	5.908	0.707	5.344
Zinc	16.060	0.676	3.555

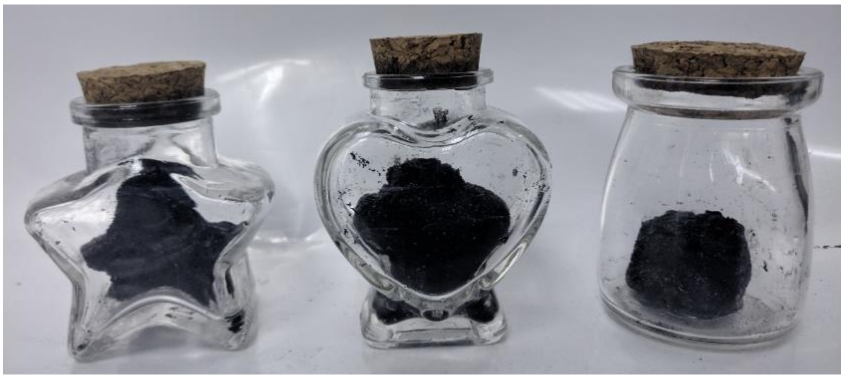

Figure S1. Photographes of ZIF-8/graphene oxide hydrogels, related to Figure 1. The shape controlled synthesis of hydrogels using reactors of various shapes. This indicates graphene hydrogels could be shape-controlled as desired.

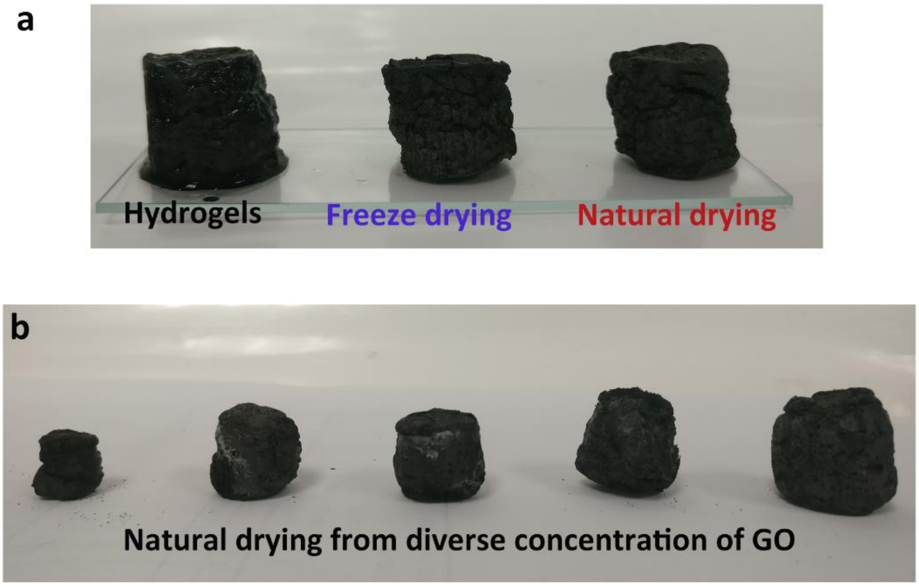

Figure S2. Photographes of ZIF-8/rGA, related to Figure 1. (a) Photo image of as-prepared ZIF-8/graphene hydrogels, ZIF-8/graphene aerogel via freeze drying, and via natural drying. Material characterization of in-situ ZIF-8/rGA composites from naturally dry. (b) Photo image of ZIF-8/rGA via freeze drying derived from diverse concentration of GO (from left to right: 1.0 mg mL^{-1}, 2.0 mg mL^{-1}, 3.0 mg mL^{-1}, 4.0 mg mL^{-1}, 5.0 mg mL^{-1}). The natural drying aerogel displayed no obvious shrinkage compared to freeze drying aerogel.

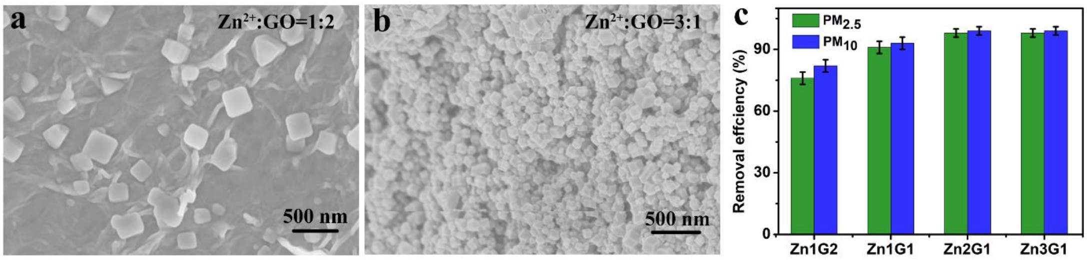

Figure S3. Removal efficiency of aerogel with diverse ration, related to Figure 3. SEM images in-situ ZIF-8 decorating on rGA with (a) low Zn^{2+}:GO ratio, and (b) high Zn^{2+}:GO ratio. In-situ process with the low Zn^{2+} proportion lead to uneven distribution of ZIF-8 while high Zn^{2+} proportion result in excessive ZIF-8 particles. (c) Particle removal effcienfy of diverse Zn^{2+}:GO ratio, the Zn1G2 represent 1:2.

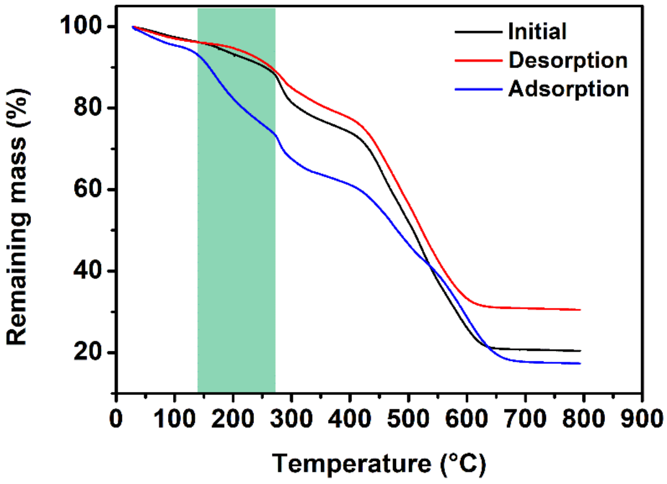

Figure S4. Comparasion of normalized remaining mass, related to Figure 5. Normalized remaining mass of initial aerogel, aerogel with PM adsorption, and aerogel with PM desorption by washing. Green fraction exhibit the difference of remaining mass after adsorping PM.

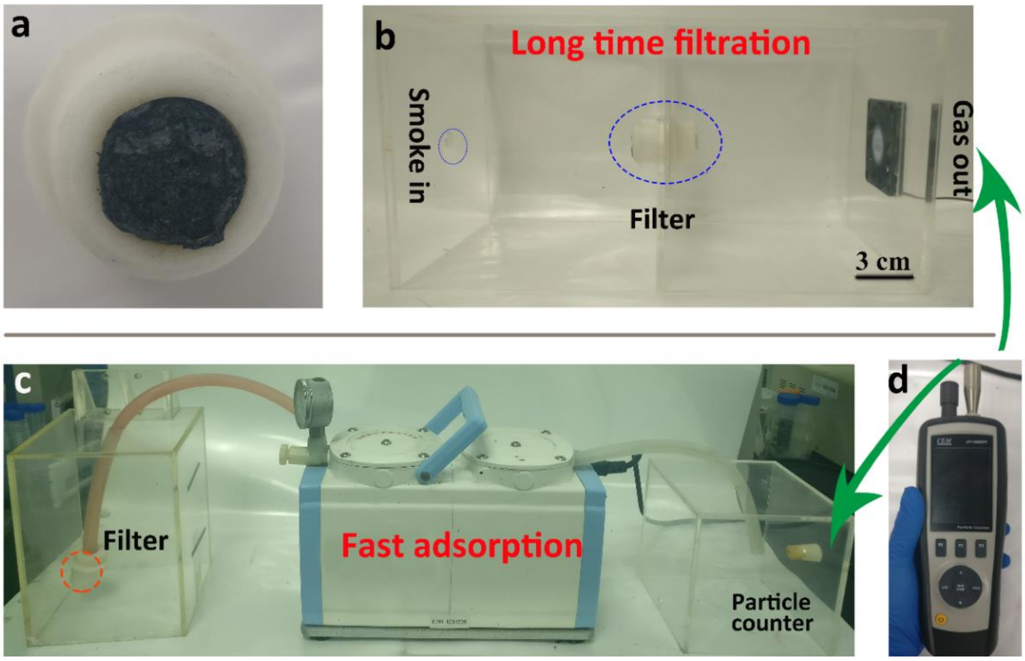

Figure S5. Photograph of PM removal device, related to Figure 3. Photo images of (a) ZIF-8/rGA filter during PM removal test through (b) home-built device. (c) Photo images of filters during fast adsorption of PM test system through home-built simulated device, (d) PM particle counter.

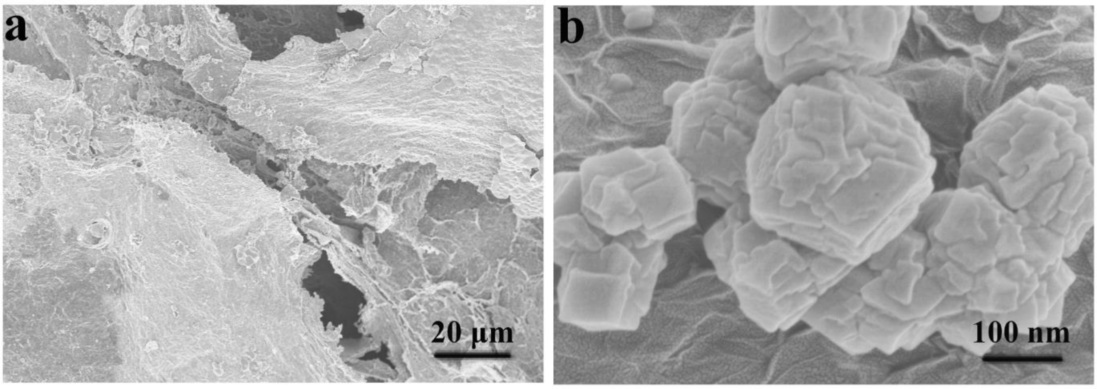

Figure S6. SEM images of PM@ZIF-8/rGA, related to Figure 3. (a) Low magnified and (b) high magnified view of PM pollution adsorbed on ZIF-8/rGA. The PM particle could be adsored on the surface of aerogel or MOFs.

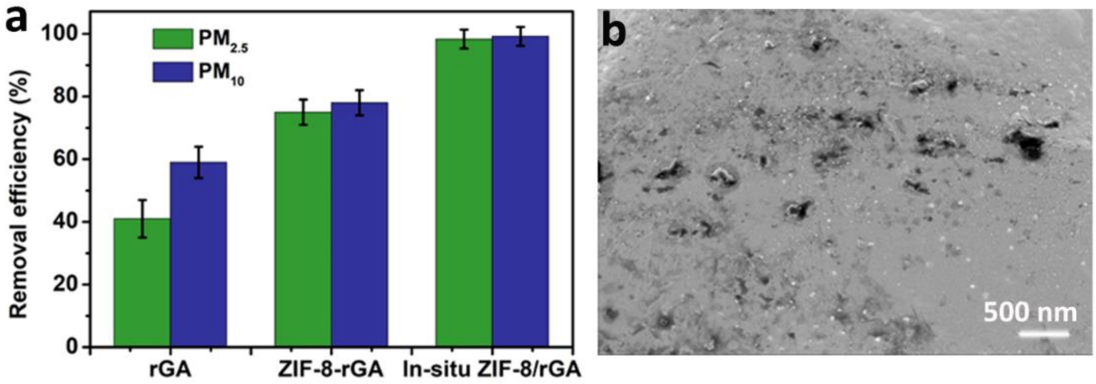

Figure S7. Removal efficiency of diverse samples, related to Figure 3. (a) PM Removal efficiency of rGA, blending ZIF-8 and rGA, in-situ ZIF-8 on rGA, (b) SEM image of PM attached on rGA surface.

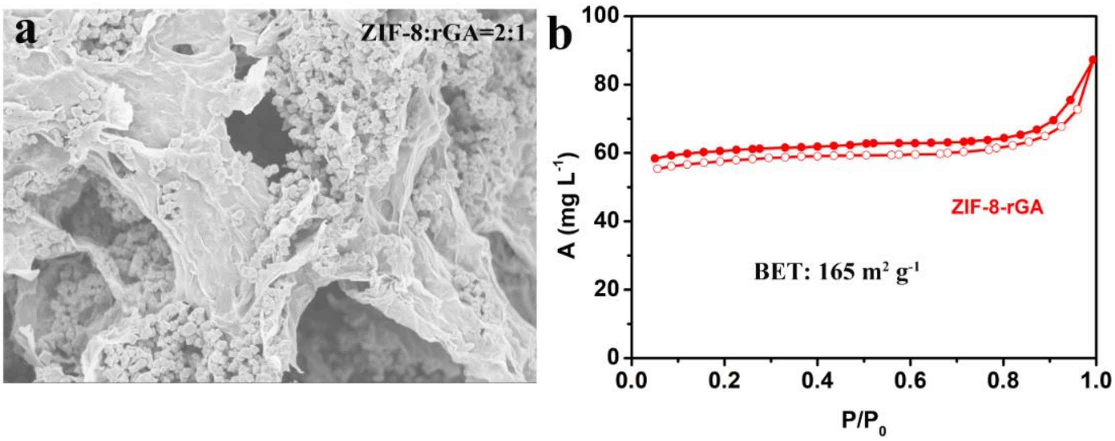

Figure S8. Specific surface area of ZIF-8/rGA, related to Figure 2. (a) SEM and (b) N_2 isotherms at 77 K of the composited aerogel by directly one-step blending ZIF-8 and rGA. The aerogels fabricated by blending show lower BET than in-situ ZIF-8/rGA.

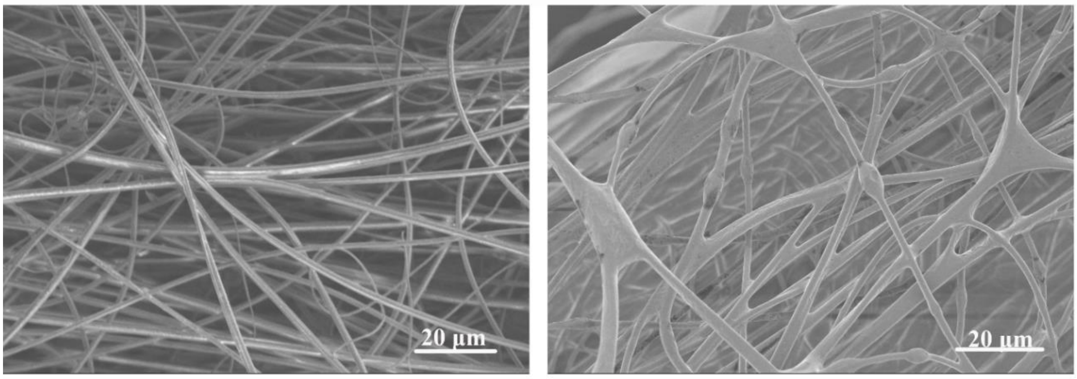

Figure S9. SEM images of PVP nanofiber, related to Figure 3. PVP nanofiber@fabric (left) and PM adsorbed on the PVP nanofiber@fabric (right).

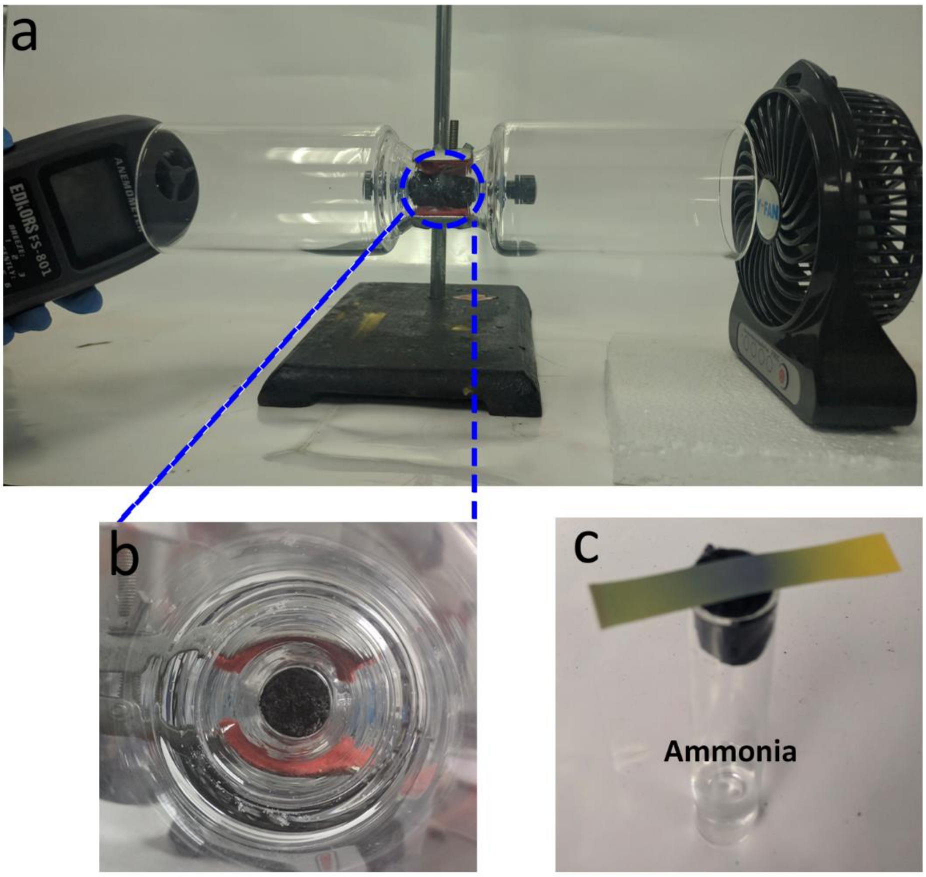

Figure S10. Air permeability of as-prepared samples, related to Figure 6. (a-b) Air permeability test of ZIF-8/rGA by a fan and an anemograph (EDKORS, FS-801), (c) air permeability test through ammonia and pH-indicator papers. The fast change of the papers indicate air flow through ZIF-8/rGA rapid.

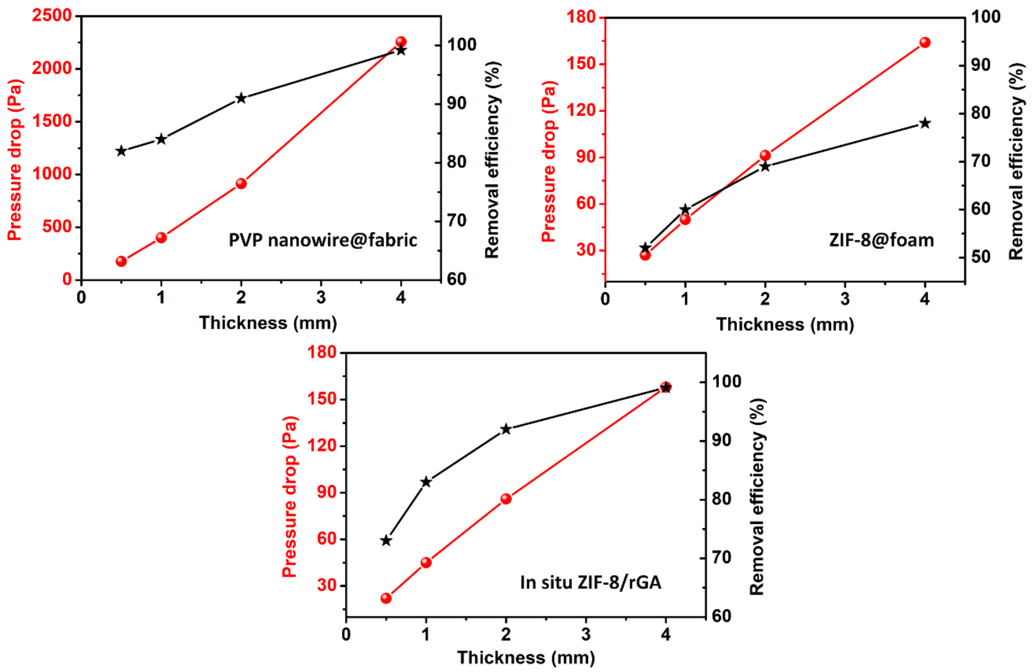

Figure S11. Pressure drop and removal efficiency, related to Figure 3. Pressure drop and removal efficiency of diverse materials in the large air flow condition.
